# Supplementary material for: Genome-Wide Identification and Expression Analysis of Cytokinin Response Regulator (RR) Genes in the Woody Plant Jatropha curcas and Functional Analysis of JcRR12 in Arabidopsis
Source: Int J Mol Sci. 2022 Sep 27;23(19):11388. doi: 10.3390/ijms231911388 (PMC9570446; doi:10.3390/ijms231911388)
Supplement: Supplementary file 1 [file ijms-23-11388-s001.zip › Text S1. Predicted amino acid sequences for the 14 Jatropha curcas response regulators (RRs).pdf]

**Supplementary Text S1** Predicted amino acid sequences for the 14 *J. curcas* response regulators (RRs)

>JcRR4 | Type-A | Gene ID: 105649825
MSSNSIASNRWMSEKMDGFDPSPNSNSDNEEEGVHVLAVDDSLVDRKVIERLLKISSCKVTAVDS
GWRALKLLGLLDEEDKSSSSSSSSSAGFDVLKVDLIITDYCMPGMTGYELLKKIKESTTFREIPVVI
MSSENVVARIDRCLEEGAEDFIVKPVKLSDVKRIRDYMASREVRSVQNQEERSSSNINKRKLQECF
DLSLSSSPPSISSSSSSSLSYPSRSPSLTPSPPSLFSSSAPCSPSSLDSPTRRLMTGFD

>JcRR7 | Type-A | Gene ID: 105640448
MAVAGEILRRSLAEEVGVSNGSSSCSEELHVLAVDDSFVDRKVIERLLRISSCKVTAVESGTRALQ
YLGLDGEKSSVGFNDLKVNLIMTDYSMPGMTGYELLKKIKESSAFREIPVVIMSSENILTRIDRCLE
EGAEEFIVKPVKLSDVKRLKDFIMKGEGEEEKRKRKILKRKLQDGVFSLSSSDLAVELPSPSRSSTIC
MPKRPKLVNRD

>JcRR8 | Type-A | Gene ID: 105638952
MEKKPSDIDEDIENQEEERQQQHFHVLAVDDSVIDRKLLERLLRNSSYQVTCVDSGNKALEYLGL
LDNLENPSTDSSSSQSTQQEGMKVNLIMTDYCMPGMSGYDLLKRVKGSSWKDVPVVVMSSENIP
SRISMCLEGGAEEFLLKPLQLSDMQKLQPHLLKSLDQSCKEAIDEKNGIEMEKNNSSNNNNNNNN
NNSKRKAMSESEEPPERRPKMGLAVVQI

>JcRR9a | Type-A | Gene ID: 105634414
MSTATESKFHVLAVDDSLLDRKLIERLLKISSYQVTTVDSGSKALQFLGLNEDDQSNSETPSVSPNN
HQEVEVNLIITDYCMPGMTGYDLLKKIKESSSLKNIPVVIMSSENVPSRITRCLEEGAEEFFLKPVQL
SDLKKLTPHMLKTKIKNQEQENQELELELERSAIHQSQQQKQPQPPPPPPLLQTPPPPPPPPPNNHKR
KAMEEGISPDRRRPRYNDITTVV

>JcRR9b | Type-A | Gene ID: 110009224
MGMATEPQFHVLAVDDSLIDRKLIERLLKTSSYHVTAVDSGSKALEFLGLNEEEQIDSNLTSVSPPG
DHHQDVEVNLIITDYCMPGMTGYDLLRKIKESKSLKDIPVVIMSSENVPSRINRCLEEGAEEFFLKP
VQLSDVNKLKPHLMKGKAKEDEPSNKRKGMDEIHSPERTRTRYEGLEVVFD

>JcRR16 | Type-A | Gene ID: 105649171
MDGGGSREFCSSMDKILGDYGDHQPHVLAVDDNLIDRKLVEKLLKNSSCRVTTAENGLRALEYL
GLGDDERKTLEKNVSKVNLIITDYCMPGMTGYELLKKIKESSILKEVPVVIMSSENIPTRIKKCLEE
GAQMFMLKPLKLSDVKKLKFDLMNCRS

>JcRR1 | Type-B | Gene ID: 105643496
MNLSNGKGSMSTASSSVAWKAGDVVSDQFPIGLRVLVVDDDPTCLMILEKMLKTCLYKVTKCTR
AEQALSMLRENKNGYDIVISDVHMPDMDGFKLLEYIGLEMDLPVIMMSADDGKNVVMKGVTHG
ACDYLIKPVRIEALKNIWQHVVRKKKNEMKDLEQSGSVEEGDRQQRQSEDADYSSSVNEGNWRN
SKKRKDEEDEADERDDTSTLKKPRVVWSVELHQQFVAAVNQLGIDKAVPKKILELMNVPGLTRE
NVASHLQKYRLYLRRLSGVSQHQNNLSNPFISPQEATYGPLSSLNGLDLQTLAATGQIPAQSLATL
QAAGLGRSAVKARMPMPIVDQRNLFSFENPKLRFGDGQQQQLNSGKQMNLLHGIPTTMEPKQLA
NLHHSAQSLGNMNIQVNNHGGQSNSLLMQMSQSQSRGQILNETTGCLVSTLPSSAGQPVISNTIAG
GVLARNGLAENGRGTGYNPVSQSPSMLNFPLNNTAELSGSSFPLGNSPGISSLTSKGAFQEDVNSEI
KGSVGFIPSYDIFSDLNQHKSHDWELQNVGMTFIASHQTNSLESNLDVGPSVLSHQGFSSSQGSAQ
NRNISAVGKPMFSAGDATDHVNPQSVGQHVNNFFADNSVRIKSETVPDANCQAGLFPDQFGQEDL
MSALLKQQQGSVGPTENEFDFDGYPMDNIPV

>JcRR11a | Type-A | Gene ID: 105644735
MMESGFSSPRHDAFPAGLRVLVVDDDPTWLKILEKMLKKCSYDVTTCGLARDALNLLRERKDGY
DIVISDVNMPDMDGFKLLEHVGLEMDLPVIMMSVDGETSRVMKGVQHGACDYLLKPIRMKELRN
IWQHVFRKKIHEVRDIEILEGMESIPMGGRNGSDQSDDGHFFCGEDFTSTKKRKDVENKHDDKDP
GDNSTTKKARVVWSVDLHQKFVKAVNQIGFDKVGPKKILDLMNVPWLTRENVASHLQKYRLYL

SRLQKENDLKTSVGGIKHSDSPLRDSAGSFGTQNSINIQQGDAPNQSYGFPGNSFQNAEPRGHDSD
LNGIVSKNAAEPKRVLTVEVPEPCKPRNSQLEFGHSFTSPGSEVNFSAFDSNFPARFSWCEIPQIQLK
QEHNPVHIDDGFNQLSFPGQQQHIQPDYPQPAHPIVSGSSVTEREIGGSIKTKPLYDECRHNTSHVSS
TGSTINSTDVQTKTHMANHQHQTFQPISMNSSTMKSQSFNLSCVSDLESSQKSINWGMHPSTTLDD
DFQVCWFQGDCYAMNLGLHNIELPEYCDPGLITEVPTHLHDAVRFDYENLYDPTEYALI

>JcRR11b | Type-B | Gene ID: 105644733
MENGSSSPRNDAFPAGLRVLVVDDDPTWLKILSKMLNKCSYEVTTCGLAREALNLLRERKDSYDI
VISDVNMPDMDGFKLLEHIGLEMDLPVISQFFAMMSVDGGASRVIKGVQHGACDYLLKPIRMKEL
RNIWQHVFRKKINVVRDIEILEGMESIPMGVRNGSDQSDDGHFFCGEDFTSTKKRKDVENKHDDK
DPGDNSSTKKARVVWSVDLHQKFVKAVNQIGFDKVGPKKILDLMNVPCLTRESVASHLQKYRLY
LSRLQKENDLKTSIGVIMDSDSCLRDSAGSSGTQKSVNKQQSDGNDLKTSVSVIKDSDLPLRDSAD
GFGTQKSINMKQSDASNYSYGFPGQPCFKYNYPSSDSCQSEGTQSSPDILHYGVLQDSFLLHNVEP
KSYGSDQEETISMADPWKDDGVATVPEPEFRNRRFLFHKIYNQNQTEN

>JcRR11L | Type-B | Gene ID: 105644734
MVENSFSHGRNNALPAGLRVLVVHADAIWVIILSKMLKQCYYEVTTCGLARDALNLLRERKDGY
DIVISDVNMPDMDGFKLLEHVGLEMDLPVIMMSVDGEKSKLMKGIQHGASDYLLKPIRMEQLRN
LWQYVLLTEKLDKVRDIEIVAGMESIQMTRNGEDLSTSAKKRRDPGDISFIEKARVVWSADLHRK
FVQAVCQIGLDKIGPQKILDLMDVPWLTREHVATHLRKYRVYFNRLQKENYLRTSVGGIKHSDSA
WKVSTGSFSTQNSINMQQSYAYASNNSNGFPGQPRFKYNFPPSNSSESVGSNKLRKDFWYDSLLL
YNVETRGYESDEEEIVSLPVGPSTISSSTDLSDASMSSNSWNSQTGFSNSSTSSGSDVNNMAAFDST
FLFHSIKT

>JcRR12 | Type-B | Gene ID: 105637574
MTVEQGIGEAKDQFPIGMRVLAVDDDPTCLLLLETLLRRCQYHVTVTNQAVSALELLRANKNKF
DLVISDVHMPDMDGFKLLELVGLEMDLPVIMLSANGDPKLVMKGISHGACDYLLKPVRMEELKN
IWQHVIRRKFDNKDRNNFDNQDRTQYGNGEATTDQKINKKRKDQNEDEDDDRDENGHENEDPT
TQKKPRVVWSVELHRKFVAAVNQLGIDKAVPKKILDLMNVEKLTRENVASHLQKYRLYLKRIST
VANQQANMVAALGSTDASYLQIGSANGLGFHNLAGTGQFHNATFRSLPPSGMLGRLNSSAALGM
RGLPSPGVIQLGHLQTTGHLNNSQGHFQPIGHAGNDGTVLQGMPMALELDQIQANKGVAYIRELP
TDINNTGAFSVSNGFPDTKTMAGSSNNPFLGVSNKPLMLEGNAQGAQDVQKFVKQTSLSVTSLDS
GISTHFPDNRTCNDSWSNAVQSTGVQSNSFSLNDCFKQSTLHPNNIRDRMSTTALQSANNPDVSSIS
TLPIDLHDSKLDLQCQVTSIRNNSGQIINNAPQGWDDQRQDATFQTNAVCSSVNSGVPIPGVAIPM
GQNFDPNNAIFHRTTSFNSERQSNFIDSSLMKHNEVENLAMETLIRSNEGYGVGQQKPQGSYVSNN
FGSLEDLASVMVKQEQDKVTFAEGEFGFGAYSLRTCI

>JcRR14 | Type-B | Gene ID: 105634128
MAALQRVASSVSATASSYGSCKGAATAAADVVASDQFPAGLRVLVVDDDTTCLKIVEQMLRRCL
YNVTTCSQAKVALNLLRERKGCYDVVLSDVHMPDMDGYKLLEQVGLEMDLPVIMMSADGRTSA
VMRGIRHGACDYLIKPIREEELKNIWQHVVRKKWHENKEHEHSGSLEDNDRHKRGNDDADHASS
VNEGTEGFLKCQKKRSSPKDEDDAEQENDDPSTSKKPRVVWSVELHQQFVSAVNQLGIDKAVPK
RILELMNVPGLTRENVASHLQVEQEKQLVPLMRSSLPSDTLILLGKDFDSGSSRPLLVLLFLCDFCL
QYFAAIAGGGCITEVLTLLMIVDPASKKSPCLRHPKFRLYLKRLSGVAQQGGISNTFCGPLDSNGK
LNSLGRFDIQALAASGQIPPQTLAALHAELFGRPTSSLVTTMDQPALLQASIQGPKCIPVEHGVAFG
QPLVKCQPSISKHFPKNIVSVEDVASGFGAWPSNNLGTVGPNTNLGCITTQNGNMLMDILHQQQQ
PQQQSIPETGRSINVQPSCLVVPSQSSASFQAGNCPASINQNCNFNRGAVIDYSLLSSQSNNTNIGHI
RDGDLKNTGVVSGYSAPGSISPTSSCSVNADSGITRQVQNSTTTFGASRHMPGLAPNICDFQGSYG
TKLGEVLDQGPLRNLGFVGKGTSIPTRFAVDEFEVPISNLSHGKVYLENNGNKVKQEPNLDFTDNA
RIGMPVLQQFHPNDLMSVFTE

>JcRR18 | Type-B | Gene ID: 105633481
MVVEDKRSGLVNEDKFPVGMRVLAVDDDPICLKVIENLLRKCQYQVTTTNQAITALNMLRENRN
KYDLVISDVNMPDMDGFKLLELVGLEMDLPVIMLSAHSDRELVYKGVTHGAVDYLLKPVRIEEL
KNIWQHVIRRKKLQTKDQNRSPNQEKPCDGAGEGGQGLSSSGSADQNGKANRKRKDQDEDEDEE
GEENINENEEPGSQKKPRVVWSVELHRKFVAAVNQLGLDKAVPKKILDLMNVEGLTRENVASHL

QKYRLYLKRISNAASQQANMVAAFGAKDPSYLRMGSLDGFGDFRTLTGPGRLSSTSISSYPSGGM
LGRLNSPAGLTLRGIASSGLLQPGHSPALSNSVNTLGKLQPALLPSSQGANLFPGVPSSLEPNQLQG
KSNAHIGDFNRNDDTSGFTLATSFSDARVTPGSLGNTVSSSISNPLMLQVNPQQNQSRGAFATQST
LSLPSMNQEPFDVGVQGSSNFLDHSRCNENWQGAVQLSKFSTNPLPLSEPFSHDPLSASNLRDSISS
TSSQIGNSPNVFSSSSALAAPLDSRVDMQGQASLIGNVFQNMNYNSRQRWDEHSQDYHSNLNNSI
SNINPLVSDNGVVGTLNQSIDQRKKFDASIVGQLNNVTPSTFQHPEVEKSSLDPKMRSNEDYLLEQ
MKSQNSFAQNNYESLDDIMNAMIKRQEQNETMLMDGEFGFDAYSLGSCI

>JcRR21 | Type-B | Gene ID: 110009834
MVKEQKTRFKLAMAATNLPDMDTIAFLHVLNEYNIPVILMSSERSISVVIKSIAEGATFYLQKPIFF
DDLKYVWQHAYRKKKTPTDSQQDQGDAQKSTDKQIMEENKKIATEEKENKENKNNNNSTEKKS
RILWTPELHMKFTAAISELGDKKARPKPILEIMKVPNLTQRQVASHLQKYKSQVQRICEAGTANLP
ALSKPYNNFYARILEKLDNKTTPNCGFRIPSGKTPELNYLKGQCMTIPSGKETNEFKVPKAVSMDG
IEKLASVEVSNAKTAIDQPYANNSLPNFDEWSRELEMMPILDPSMEVPELDKILTSSDDASNNMEA
PRNISSPPASTVNEDQPSLLKFADDLLNIMEEEPATGEPNPSDVDRYCEWLIS
