# Supplementary material for: Genome-Wide Identification and Expression Analysis of Cytokinin Response Regulator (RR) Genes in the Woody Plant Jatropha curcas and Functional Analysis of JcRR12 in Arabidopsis
Source: Int J Mol Sci. 2022 Sep 27;23(19):11388. doi: 10.3390/ijms231911388 (PMC9570446; doi:10.3390/ijms231911388)
Supplement: Supplementary file 1 [file ijms-23-11388-s001.zip › Text S2. Predicted amino acid sequences for the 21 Arabidopsis response regulators (RRs).pdf]

Supplementary Text S2 Predicted amino acid sequences for the 21 *Arabidopsis* response regulators (RRs)

>ARR1 | Type-B | Gene ID: 820940
MMNPSHGRGLGSAGGSSSGRNQGGGGETVVEMFPSGLRVLVVDDDPTCLMILERMLRTCLYEVT
KCNRAEMALSLLRKNKHGFDIVISDVHMPDMDGFKLLEHVGLEMDLPVIMMSADDSKSVVLKGV
THGAVDYLIKPVRMEALKNIWQHVVRKRRSEWSVPEHSGSIEETGERQQQHRGGGGGAAVSGG
EDAVDDNSSSVNEGNNWRSSSRKRKDEEGEEQGDDKDEDASNLKKPRVVWSVELHQQFVAAVN
QLGVEKAVPKKILELMNVPGLTRENVASHLQKYRIYLRRLGGVSQHQGNLNNSFMTGQDASFGP
LSTLNGFDLQALAVTGQLPAQSLAQLQAAGLGRPAMVSKSGLPVSSIVDERSIFSFDNTKTRFGEG
LGHHGQQPQQQPQMNLLHGVPTGLQQQLPMGNRMSIQQQIAAVRAGNSVQNNGMLMPLAGQQ
SLPRGPPPMLTSSQSSIRQPMLSNRISERSGFSGRNNIPESSRVLPTSYTNLTTQHSSSSMPYNNFQPE
LPVNSFPLASAPGISVPVRKATSYQEEVNSSEAGFTTPSYDMFTTRQNDWDLRNIGIAFDSHQDSES
AAFSASEAYSSSSMSRHNTTVAATEHGRNHQQPPSGMVQHHQVYADGNGGSVRVKSERVATDT
ATMAFHEQYSNQEDLMSALLKQEGIAPVDGEFDFDAYSIDNIPV

>ARR2 | Type-B | Gene ID: 827297
MVNPGHGRGPDSGTAAGGSNSDPFPANLRVLVVDDDPTCLMILERMLMTCLYRVTKCNRAESAL
SLLRKNKNGFDIVISDVHMPDMDGFKLLEHVGLEMDLPVIMMSADDSKSVVLKGVTHGAVDYLI
KPVRIEALKNIWQHVVRKKRNEWNVSEHSGGSIEDTGGDRDRQQQHREDADNNSSSVNEGNGRS
SRKRKEEEVDDQGDDKEDSSSLKKPRVVWSVELHQQFVAAVNQLGVDKAVPKKILEMMNVPGX
TRENVASHLQKYRIYLRRLGGVSQHQGNMNHSFMTGQDQSFGPLSSLNGFDLQSLAVTGQLPPQS
LAQLQAAGLGRPTLAKPGMSVSPLVDQRSIFNFENPKIRFGDGHGQTMNNGNLLHGVPTGSHMRL
RPGQNVQSSGMMLPVADQLPRGGPSMLPSLGQQPILSSSVSRRSDLTGALAVRNSIPETNSRVLPTT
HSVFNNFPADLPRSSFPLASAPGISVPVSVSYQEEVNSSDAKGGSSAATAGFGNPSYDIFNDFPQHQ
QHNKNISNKLNDWDLRNMGLVFSSNQDAATATATAAFSTSEAYSSSSTQRKRRETDATVVGEHG
QNLQSPSRNLYHLNHVFMDGGSVRVKSERVAETVTCPPANTLFHEQYNQEDLMSAFLKQEGIPSV
DNEFEFDGYSIDNIQV

>ARR3 | Type-A | Gene ID: 842288
MAKDGGVSCLRRSEMIGIGIGELESPPLDSDQVHVLAVDDSLVDRIVIERLLRITSCKVTAVDSGW
RALEFLGLDDDKAAVEFDRLKVDLIITDYCMPGMTGYELLKKIKESTSFKEVPVVIMSSENVMTRI
DRCLEEGAEDFLLKPVKLADVKRLRSYLTRDVKVAAEGNKRKLTTPPPPPPLSATSSMESSDSTVE
SPLSMVDDEDSLTMSPESATSLVDSPMRSPGLA

>ARR4 | Type-A | Gene ID: 837587
MARDGGVSCLRRSEMMSVGGIGGIESAPLDLDEVHVLAVDDSLVDRIVIERLLRITSCKVTAVDSG
WRALEFLGLDNEKASAEFDRLKVDLIITDYCMPGMTGYELLKKIKESSNFREVPVVIMSSENVLTR
IDRCLEEGAQDFLLKPVKLADVKRLRSHLTKDVKLSNGNKRKLPEDSSSVNSSLPPPSPPLTISPESS
PPLTVSTESSDSSPPLSPVEIFSTSPLSSPIDDEDDDVLTSSSEESPIRRQKMRSPGLD

>ARR5 | Type-A | Gene ID: 823965
MAEVLRPEMLDISNDTSSLASPKLLHVLAVDDSMVDRKFIERLLRVSSCKVTVVDSATRALQYLG
LDGENNSSVGFEDLKINLIMTDYSMPGMTGYELLKKIKESSAFREIPVVIMSSENILPRIDRCLEEGA
EDFLLKPVKLADVKRLRDSLMKAEERAFKNIMHKRELEANDIYSQLKRAKI

>ARR6 | Type-A | Gene ID: 836412
MAEVMLPRKMEILNHSSKFGSPDPLHVLAVDDSHVDRKFIERLLRVSSCKVTVVDSATRALQYLG
LDVEEKSVGFEDLKVNLIMTDYSMPGMTGYELLKKIKESSAFREVPVVIMSSENILPRIDRCLEEGA
EDFLLKPVKLSDVKRLRDSLMKVEDLSFTKSIQKRELETENVYPVHSQLKRAKI

>ARR7 | Type-A | Gene ID: 838487
MAVGEVMRMEIPAGGDLTVTTPELHVLAVDDSIVDRKVIERLLRISSCKVTTVESGTRALQYLGLD
GGKGASNLKDLKVNLIVTDYSMPGLSGYDLLKKIKESSAFREVPVVIMSSENILPRIQECLKEGAEE

FLLKPVKLADVKRIKQLIMRNEAEECKILSHSNKRKLQEDSDTSSSSHDDTSIKDSSCSKRMKSESE
NLFSLL

>ARR8 | Type-A | Gene ID: 818730
MVMETESKFHVLAVDDSLFDRKMIERLLQKSSCQVTTVDSGSKALEFLGLRVDDNDPNALSTSPQ
IHQEVEINLIITDYCMPGMTGYDLLKKVKESAAFRSIPVVIMSSENVPARISRCLEEGAEEFFLKPVK
LADLTKLKPHMMKTKLKKESEKPVAIEEIVVSKPEIEEEEEESSVIEILPLHQEIESEQLEPMLSSNKR
KAMEEVVSTDRSRPKYNDITTSV

>ARR9 | Type-A | Gene ID: 824871
MGMAAESQFHVLAVDDSLFDRKLIERLLQKSSCQVTTVDSGSKALEFLGLRQSTDSNDPNAFSKA
PVNHQVVEVNLIITDYCMPGMTGYDLLKKVKESSAFRDIPVVIMSSENVPARISRCLEEGAEEFFLK
PVRLADLNKLKPHMMKTKLKNQKLEEIETTSKVENGVPTAVADPEIKDSTNIEIEILPLQQDLLLVQ
QEEQTLSINNKRKSVEEGISTDRARPRFDGIATAV

>ARR10 | Type-B | Gene ID: 829322
MTMEQEIEVLDQFPVGMRVLAVDDDQTCLRILQTLLQRCQYHVTTTNQAQTALELLRENKNKFD
LVISDVDMPDMDGFKLLELVGLEMDLPVIMLSAHSDPKYVMKGVKHGACDYLLKPVRIEELKNI
WQHVVRKSKLKKNKSNVSNGSGNCDKANRKRKEQYEEEEEEERGNDNDDPTAQKKPRVLWTHE
LHNKFLAAVDHLGVERAVPKKILDLMNVDKLTRENVASHLQKFRVALKKVSDDAIQQANRAAID
SHFMQMNSQKGLGGFYHHHRGIPVGSGQFHGGTTMMRHYSSNRNLGRLNSLGAGMFQPVSSSFP
RNHNDGGNILQGLPLEELQINNNINRAFPSFTSQQNSPMVAPSNLLLLEGNPQSSSLPSNPGFSPHFE
ISKRLEHWSNAALSTNIPQSDVHSKPDTLEWNAFCDSASPLVNPNLDTNPASLCRNTGFGSTNAAQ
TDFFYPLQMNQQPANNSGPVTEAQLFRSSNPNEGLLMGQQKLQSGLMASDAGSLDDIVNSLMTQ
EQSQSDFSEGDWDLDGLAHSEHAYEKLHFPFSLSA

>ARR11 | Type-B | Gene ID: 843096
EKSGFSPVGLRVLVVDDDPTWLKILEKMLKKCSYEGPTCGLAREALRLLRERKDGYDIVISDVNM
PDMDGFKLLEHVGLELDLPVISVSVDGETSRVMKGVQHGACDYLLKPIRMKELKIIWQHVLRKKL
QEVRDIEGCGYEGGADWITRYDEAHFLGGGEDVSFGKKRKDFDFEKKLLQDESDPSSSSSKKARV
VWSFELHHKFVNAVNQIGCDHSTGPKKILDLMNVPWLTRENVASHLQVYRLYLSRLEKGKELKC
YSGGVKNADSSPKDVEVNSGYQSPGRSSYVFSGGNSLIQKATEIDPKPLASASLSDLNTDVIMPPKT
KKTRIGFDPPISSSAFDSLLPWNDVPEVLESKPVLYENSFLQQQPLPSQSSYVANSAPSLMEEEMKP
PYETPAGGSSVNADEFLMPQDKIPTVTLQDLDPSAMKLQEFNTEAILRSLNWELPESHHSVSLDTD
LDLTWLQGERFLANTGLQFQDYSSSPSLLSELPAHLNWYGNERLPDPDEYSFMVDQGLFIS

>ARR12 | Type-B | Gene ID: 817056
MTVEQNLEALDQFPVGMRVLAVDDDQTCLKILESLLRHCQYHVTTTNQAQKALELLRENKNKFD
LVISDVDMPDMDGFKLLELVGLEMDLPVIMLSAHSDPKYVMKGVTHGACDYLLKPVRIEELKNI
WQHVVRSRFDKNRGSNNNGDKRDGSGNEGVGNSDQNNGKGNRKRKDQYNEDEDEDRDDNDDS
CAQKKQRVVWTVELHKKFVAAVNQLGYEKAMPKKILDLMNVEKLTRENVASHLQKFRLYLKRI
SGVANQQAIMANSELHFMQMNGLDGFHHRPIPVGSGQYHGGAPAMRSFPPNGILGRLNTPSGIGV
RSLSSPPAGMFLQNQTDIGKFHHVSSLPLNHSDGGNILQGLPMPLEFDQLQTNNNKSRNMNSNKSI
AGTSMAFPSFSTQQNSLISAPNNNVVVLEGHPQATPPGFPGHQINKRLEHWSNAVSSSTHPPPPAH
NSNSINHQFDVSPLPHSRPDPLEWNNVSSSYSIPFCDSANTLSSPALDTTNPRAFCRNTDFDSNTNV
QPGVFYGPSTDAMALLSSSNPKEGFVVGQQKLQSGGFMVADAGSLDDIVNSTMKQEQSQGDLSG
GDLGYGGFSSLRTCI

>ARR13 | Type-B | Gene ID: 817249
MAFAQSVYNQSSVLKINVMVVDDNRVFLDIWSRMLEKSKYREITVIAVDYPKKALSTLKNQRDNI
DLIITDYYMPGMNGLQLKKQITQEFGNLSVLVMSSDPNKEEESLSCGAMGFIPKPIAPTDLPKIYQF
ALTYKRNGKSTLSTEQNQKDANVSVPQQIMLVPEQAYVLKTKKKNCSSKSDTRTVNSTNVSHVST
NGSRKNRKRKPKGGPSDDGESLSQPPKKKKIWWTNPLQDLFLQAIQHIGYDKVVPKKILAIMNVP
YLTRENVASHLQKYRLFVKRVVHQGRFSMLSDRGKDSMFRQTHIKEPYVNYYTPSTSWYETSLN
NRSFYSESVHGHSRLLSEAREPVRYNQMSYNYMNRNISFENQPSQNEETRTVFEPPVMANKISQTS
QVLGFGQLGPSAISGHNFNTNMMSSYGSLTPNQPGTSHFSYGMQSVLNNENATYNPQPPANATTQ

PNLDELPQLENLNLYNDLGNTSELPYNISNFQSDDNKKQGEEDGDWTFVNINQDQSNGESSNTIAT
PETNTPNFNINPNQNQGQAVPEFTDWSFLDQQELVDDDFMNSLFNNDMN

>ARR14 | Type-B | Gene ID: 814707
MPINDQFPSGLRILVVDDDTSCLFILEKMLLRLMYQVTICSQADVALTILRERKDSFDLVLSDVHMP
GMNGYNLLQQVGLLEMDLPVIMMSVDGRTTTVMTGINHGACDYLIKPIRPEELKNIWQHVVRRK
CVMKKELRSSQALEDNKNSGSLETVVVSVSECSEESLMKCRNKKKKKKRSVDRDDNEDDLLLDP
GNSKKSRVVWSIELHQQFVNAVNKLGIDKAVPKRILELMNVPGLSRENVASHLQKFRLYLKRLSG
EASQSNDSESTKRYENIQALVSSGQLHPQTLAALFGQPIDNHHSASFGVWIPNDNLGRSQNEHFSV
DVSSASNRPVSVAVHGLSSSANFRQRGDVNNNRIRQGYGSNVNEESWILERSSRQR

>ARR15 | Type-A | Gene ID: 843828
ALRDLSSSLSSSSSPELHVLAVDDSFVDRKVIERLLKISACKGTTVESGTRALQYLGLDGDNGSSGL
KVLKVNLIVTDYSMPGLTGYELLKKIKVSSALREIPVVIMSSENIQPRIEQYMIEGAEEFLLKPVKLA
DVKRLKELIMRGGEAEEGKTKKLSPKRILQNDIDSSPSSSSTSSSSSSHDVSSLDDDTPSSKRIKLESR
G

>ARR16 | Type-A | Gene ID: 818662
MNSSGGSCSSLMDVVAYDHHLHHGHDEELHVLAVDDNLIDRKLVERLLKISCCKVTTAENALRA
LEYLGLGDQNQHIDALTCNVMKVSLIITDYCMPGMTGFELLKKVKQESSNLREVPVVIMSSENIPT
RINKCLASGAQMFMQKPLKLADVEKLKCHLMNCRS

>ARR17 | Type-A | Gene ID: 824805
MNKGCGSGSDSCLSSMEEELHVLAVDDNLIDRKLVERILKISSCKVTTAENGLRALEYLGLGDPQQ
TDSLTNVMKVNLIITDYCMPGMTGFELLKKVKESSNLKEVPVVILSSENIPTRINKCLASGAQMFM
QKPLKLSDVEKLKCHLLNCRS

>ARR18 | Type-B | Gene ID: 835920
MEFGSTEDGRHDKFPVGMRVLAVDDNPTCLRKLEELLLRCKYHVTKTMESRKALEMLRENSNMF
DLVISDVEMPDTDGFKLLEIGLEMDLPVIMLSAHSDYDSVMKGIIHGACDYLVKPVGLKELQNIW
HHVVKKNIKSYAKLLPPSESDSVPSASRKRKDKVNDSGDEDDSDREEDDGEGSEQDGDGSGTRKK
PRVVWSQELHQKFVSAVQQLGLDKAVPKKILDLMSIEGLTRENVASHLQKYRLYLKKIDEGQQQ
NMTPDAFGTRDSSYFQMAQLDGLRDFTAARQIPSSGLLSRSHLTKLQPPMYSSINLQGMNSSSFIQ
QGHHQNSSNSANPFGTYHSTLSPRIQNVNLFQRTSSPLEPLQFPRSKSYIGDFKGLGDRAIGGSFLDT
CMPFGSSSTSLPSASTNPLMLQANYTQPLHIASDGIQPCIEGTPSNSASPNISFQGLSRFPGHSWQGN
LNTTRFPPSSLPLNLAFLPDQVTCAGNNLGDCTSLVSAENPGGEMQCDPQLLGGFMQNVNPLGGQ
KWEQQNCTMLNNPFGNIEYPLPADNMVFRDNNSTRSKGLDESLMNPIDNSQEYVGKATTMLDPE
MKSGKPENDNQHDVFDDIMNEMMKQEENNGMVPVATRFGFDSFPPP

>ARR19 | Type-B | Gene ID: 841342
MLVGKISGYEDNTRSLERETSEITSLLSQFPGNTNVLVVDTNFTTLLNMKQIMKQYAYQVSIETDA
EKALAFLTSCKHEINIVIWDFHMPGIDGLQALKSITSKLDLPVVIMSDDNQTESVMKATFYGACDY
VVKPVKEEVMANIWQHIVRKRLIFKPDVAPPVQSDPARSDRLDQVKADFKIVEDEPIINETPLITWT
EEIQPVQSDLVQANKFDQVNGYSPIMNQDNMFNKAPPKPRMTWTEVIQPVQSNLVQTKEFGQLN
DYSQIMNQDSMYNKAATKPQLTWTEEIQPVQSGLVQANEFSKVNGYSQSMNQDSMFNKSATNPR
LTWNELLQPVQSDLVQSNEFSQFSDYSQIMNEDNMFNKAAKKPRMTWSEVFQPVQSHLVPTDGL
DRDHFDSITINGGNGIQNMEKKQGKKPRKPRMTWTEELHQKFLEAIEIIGGIEKANPKVLVECLQE
MRIEGITRSNVASHLQVKKKTHTLNIKHRINLEENQIPQQTQGNGWATAYGTLAPSLQGSDNVNTT
IPSYLMNGPATLNQIQQNQYQNGFLTMNNNQIITNPPPPLPYLDHHHQQQHQSSPQFNYLMNNEEL
LQASGLSATDLELTYPSLPYDPQEYLINGYNYN

>ARR20 | Type-B | Gene ID: 825441
MSVFSNILDENSRNLRNEIPCDDGIASPINDDDEEFLTKSNRVLLVGADSNSSLKNLMTQYSYQVT
KYESGEEAMAFLMKNKHEIDLVIWDFHMPDINGLDALNIIGKQMDLPVVIMSHEYKKETVMESIK
YGACDFLVKPVSKEVIAVLWRHVYRKRMSKSGLDKPGESGTVESDPDEYDDLEQDNLYESNEEG
SKNTCDHKEEKSPTKKPRMQWTPELHHKFEVAVEKMGSLEKAFPKTILKYMQEELNVQGLTRNN

VASHLQKYRQSSKKTCTPQEPQEDFVWGNAGPDVTLAASKTLLSSHATPSYLINNQAAPRGSYFM
NNIPYPSTSCLPVNNNNCFMTNPSTYIDQFQHQLQQQQHQQYQSTLNSISAMLTKQESRHVPSSA
MENSEPLMIYNSNLPFGIDECFPPAGFNIFDQIGHN

>ARR21 | Type-B | Gene ID: 830612
MASAQSFYNQSSVLKINVMVVDDDHVFLDIMSRMLQHSKYRDPSVMEIAVIAVDDPKKALSTLKI
QRDNIDLIITDYYMPGMNGLQLKKQITQEFGNLPVLVMSSDTNKEEESLSCGAMGFIPKPIHPTDLT
KIYQFALSNKRNGKSTLSTEQNHKDADVSVPQQITLVPEQADVLKTKRKNCSFKSDSRTVNSTNGS
CVSTDGSRKNRKRKPNGGPSDDGESMSQPAKKKKIQWTDSLHDLFLQAIRHIGLDKAVPKKILAF
MSVPYLTRENVASHLQKYRIFLRRVAEQGLYSMLSDRGIDSMFRQTHIKEPYFNYYTPSTSWYDT
RLNNRSFYSKPVHGFGQSKLLSTTREPVCFNQMPYNYMNRSSTYEPHRIGSGSNLTLPIQSNLSFPN
QPSQNEERRSFFEPPVMANKIAQTSQVLGFGQLGPSAISGHNFNNNMTSRYGSLIPSQPGPSHFSYG
MQSFLNNENVTYNPQPPANATTQPNLDELPQLENLNLYNDFGNTSELPYNISNFQFDDNKHQQGE
ADPTKFELPAAKFSTELNHEDDGDWTFVNINQGQSNGETSNTIASPETNTPILNINHNQNQGQDVP
EFNDWSFLDPQELVDDDFMNSLFNNDMN
