# Supplementary material for: Genome-Wide Identification and Expression Analysis of Cytokinin Response Regulator (RR) Genes in the Woody Plant Jatropha curcas and Functional Analysis of JcRR12 in Arabidopsis
Source: Int J Mol Sci. 2022 Sep 27;23(19):11388. doi: 10.3390/ijms231911388 (PMC9570446; doi:10.3390/ijms231911388)
Supplement: Supplementary file 1 [file ijms-23-11388-s001.zip › Text S3. Predicted amino acid sequences for the 22 Populus response regulators (RRs).pdf]

Supplementary Text S3 Predicted amino acid sequences for the 22 *Populus* response regulators (RRs)

>PtRR1 | Type-A | Gene ID: 18102382
MSSNSIASNRWMSEKMDGFDPSPNSNSDNEEEGVHVLAVDDSLVDRKVIERLLKISSCKVTAVDS
GWRALKLLGLLDEEDKSSSSSSSSSSAGFDVLKVDLIITDYCMPGMTGYELLKKIKESTTFREIPVVI
MSSENVVARIDRCLEEGAEDFIVKPVKLSDVKRIRDYMASREVRSVQNQEERSSSNINKRKLQECF
DLSLSSSPPSISSSSSSSLSYPSRSPSLTPSPPSLFSSSAPCSPSSLDSPTRRLMTGFD

>PtRR2 | Type-A | Gene ID: 7469072
MGSNSIVSNRWMSEKMNCLDLSPNSNSDNEEEEVHVLAVDDSFVDRKVIERLLKISSCKVTAVDS
GWGALKLLGLLDDEEEDKSSSSSSSSSSSAGFEGLKVDLIITDYCMPGMTGYELLKKIKESSSFREIPV
VIMSSENVMARIDRCLEEGAEEFIAKPVKLSDVKRLRDYYMATREIRSVQSSSNVNKRKLQESFDV
STSSSPPSISPSPSPSSASPPSLFSSSAPCSPSSLDSPTRRIKMTSFD

>PtRR3 | Type-A | Gene ID: 7466460
MEIMESGVVDTQHQEEEKHQQQKEEEGEEKHKQQRDQEGEEEEKHFHVLAVDDSFIDRKLLERL
LKVSSYQVTFVDSGDKALEYLGLLDSIDNVNATSSSSSSQSPQQEGMKVNLIMTDYCMPGMSGYD
LLKRVKGSYWKDVPVVVMSSENIPSRIRMCLEEGAEEFLLKPLQLSDVEKLQTHLLKSLDKYSSKR
IDDNSFNTDSSNITVSNKSNSNNIVSKRKALSPEIEDRRPKMKGLAVV

>PtRR4 | Type-A | Gene ID: 7462788
MATLATLATETQFHVLAVDDSLIDRKLIERLLKTSSYQVTAVDSGSKALEFLGLSSNPSSVSPDHH
HQHIEINMIITDYCMPGMTGYDLLKKIKESKYFKDIPVVIMSSENVPSRINRCLEEGAEEFFLKPVQL
SDVNKLRPHLMKGRCKEEDQPNNKRKGMEEIVNSPGRTRSRYNEGLEVVLSQ

>PtRR5 | Type-A | Gene ID: 7476846
MAVEMALATTMATETQFHVLAVDDCLIDRKLIERLLKTSSYQVTAVDSGSKALEFLGLNGENELR
DSKPASVSPDPYHQHIEINMIITDYCMPGMTGYDLLKKIKESKYFKDIPVVIMSSENVPSRINRCLKE
GAEEFFLKPVQLSDVNKLRPHLMKGRCKEEEEEEEEDQPNNKRKGMEEIVNSPDRTRTRYNDGLE
VV

>PtRR6 | Type-A | Gene ID: 7473567
MTITGDSHSQFHVLAVDDSMIDRKLIERLLKTSSYQVTAVDSGSKALKFLGLHEEDDHSNPDTVPS
VSPNDHREVEVNLIITDYCMPGMTGYDLLKKVKESSSLRDIPVVIMSSENVPSRITRWREIIKARIGC
LEEGAEEFFLKPVRLADLNRLKPHMMKTKIKNEKQEDQEKLENSEIQSEQQPQPPSQPQPQPQPQL
ESQSQPQPSPLLQQPNNNKRKAMEEGLSPDRTRPRYNGITTMV

>PtRR7 | Type-A | Gene ID: 7464108
MAITGDSLSQFHVLAVDDSLIDRKLIERLLKTSSYQVTTVDSGSKALKFLGLQEDEQSNPDTPYVSP
NNHQEMEVNLIITDYCMPGMTGYDLLKKVKESSSLRNIPVVIMSSENVPSRITRCLEEGAEEFFLKP
VRLSDLNRLKPHMMKTKIKNQKQEEQEELEIPAIQSEEQKQPVQQPSSQPQPESQPQPSALLLQPNN
NKRKAMEEGLSPDRTRPRYNGITTMV

>PtRR8 | Type-A | Gene ID: 7459242
MDGGGGSRENVMVGGAYTEEPHVLAVDDSLVDRKLVERLLKNSSCKVTTAENGLRALEYLGLG
DEKRTSLEDNVSKVNLIITDYCMPGMTGYELLKKIKESSMLKEIPVVIMSSENIPTRINKCLEEGAQ
MFMLKPLKQSDVVKLRCNLMNCRS

>PtRR9 | Type-A | Gene ID: 7494641
MAGSSSSSPSMGFDFDEKLHVLAVEAVDDGLIDRKAIERLLINSEYKVTTAENKKKAIEYLGLADG
HHTNHDLKVNLIITDYCMRGMTGYELLKRIKESPTMKGDTVVVVSSENIPTRIKGCMEEGAQEFLL
KPLQLSGVTKLSAI

>PtRR10 | Type-A | Gene ID: 7462538

MATAGEILRRSLTEEVGFSKGSVSGSEELHVLAVDDSFVDRKVIERLLKISSCKVTVVESGSRALQY
LGLDGEKSSVGFNDLKINLIMTDYSMPGMTGYELLKKIKESSAFREIPVVIMSSENILARIDRCLEEG
AEEYILKPVKLSDVKRIKDVIMGGDGEQKKRRSVRKRGREDCFYSLSQPQLVQSSSSPAFDFLPSEL
QSSSPFSTLSLSKRPKLQTR

>PtRR11 | Type-A | Gene ID: 7494641
MASSSSSLPSMEFDFDEKPHVLAVDDSLIDRKVIERLLINSTCRVTTAENGKRALEYLGLADGQHPS
HSDLKVNMIITDYSMPGMTGYELLKRIKESPTMKEIPVVVVSSENIPTRINQCMEGGAQEFLLKPLQ
LSDATKLRCHIKKLNN

>PtRR12 | Type-B | Gene ID: 18101870
MMNLANCKGSMSTATSGGVWKASDGASDQFPAGLRVLVVDDDPTCLVILEKMLRTCRYEVTKC
NRAEIALSLLRENKNGYDIVISDVHMPDMDGFKLLEQIGLEMDLPVIMMSADDGKNVVMKGVTH
GACDYLIKPIRIEALKNIWQHVVRKRKNEWKDLEQSGSVEEGGDRQQKQPEDADYSSSANEGSW
KNSKRRKDEEEEAEERDDTSALKKPRVVWSVELHQQFVAAVNQLGIDKAVPKKILELMNVPGLT
RENVASHLQKYRLYLRRLSGVSQHQNGMGSSFISPQEATYGPLSSLNGLDLQTLATAGQLPAQSL
ATLQAAGLGRSTAKPRMPMPIVDQRNLFSFENPKLRFGEGQQQHLNNGKQTNLLHGIPTTMEPKQ
LANLHHSAQSLGSMNMQFNAHAGQSSSLLMQMSQQQSRGQILNETTHSHVPRLSSSIGQPIASNAL
ASGVLTRNGLAENGRGIGFNPVSQSSTLLNFPLNTTAELTATSFPLGSAAEVPSLTSKGTFQEEISSEI
KGTGGFMPSYDIFSDLQQHRSHDWELQNVGMTFNGSQQSNSVQSNLDVAPSVLSHQGFSSCQSN
GQSRNNISVVGKPMFSAGDATEHVNAQSLEHPINTFFAENSMRVKTERVPDANPQTALFNGQFGQ
EDLMSALLKQ

>PtRR13 | Type-B | Gene ID: 7477193
MLNLGYCKGSMSTASSGGVSDQFPAGLRVLVVDDDPTCLVILEKMLRTCLYEVTKCNRAEIALSL
LRENRNGYDIVISDVHMPDMDGFKLLELIGLEMDLPVIMMSADDGKNVVMKGVTHGACDYLIKP
IRIEALKNIWQHVVRKRKNEWKDLEQSGSVEEGGDRQQKQSEDADYSSSANEGSWKNSKRRKDE
EEEADERDDTSTLKKPRVVWSVELHQQFVAAVHQLGIDKAVPKKILELMNVPGLTRENVASHLQ
KYRLYLRRLSGVSQHQSGMGNSFINPQEATYGPLSSLNGLDLQTLAAAGQIPAQSLATLQAAGLG
RSTAKPRMPMPIVDQRNLFSFENPKLRFGEGQQQHLNNGKQINLLHGIPTTMEPKQLADLHHSAQS
LGSMNMQLNAHGDQSGQSGSLLMQMSQQQSRGQILNETTSSQVPRLPSSIGQPIVTNAIASGVLAR
NGLAENGRGTGFNPVSQSSPTLNFPLNTTAELTATSFALGSAPGVPSLTSKGTFPEDISSEMKGPGG
FIMPSYDIFSELQHHRSHDWELQNVGMAFNGSQQSNSLQSNVDVASSVLAHQGFSSSQSNGQGRN
ISAVSKPIFSAGDATSHVNAQSLGQPLNTFFAENLVRVKTERVPDANLQTTLFNEQFGQEDLMSAL
LKQQQAGIGPAEIEFDFDG

>PtRR14 | Type-B | Gene ID: 7494797
MMENNGFSSPRNDSFPAGLRVLVVDDDPTWLKILEKMLKKCSYEVTTCGLARDALNLLRERKGG
YDIVISDVYMPDMDGFKLLEQVGLEMDLPVIMMSVDGETSRVMKGVQHGACDYLLKPIRMKELR
NIWQHVFRKKIHEVRDIETLERVESFQMTRNGSDQYEDGHVLCGEDLTSIKKRKHIESKHDEKDTG
DSISTKKARVVWSVDLHQKFVKAVNQIGFDKVGPKKILDMMNVPWLTRENVASHLQKYRLYLSR
LQKENDFKNPAGGIKQSDSPLRDSAGSFGSQNSINLQHNDVSNGSYRFSGSSLVVHNGDPRSHDSD
RKRLVSTPVEEPKRTLTVNVPNPCIPRSSQMEFGHPLAPPQSEVDFGALDSTFPTKYPWCGIPEIQLK
KEHNPLHLNDEFSHLPLPGQKQLTQADYPQPAPAISSAPSLTPSHNEYRSNVNHASSTAIAVDSSPI
QTKTNVANHQAIELISKSTPSLENQGFNMNSITEFESSRKNINLGMLPFTTLEEDLQVCWVPGDCY
MNLGLQNIEVLEYFDPGLITDVPVNLNDGLRFDYEFNDPTEYSLIDQSLFIA

>PtRR15 | Type-B | Gene ID: 7457808
MAALQRVAPSLGTSASTYGSCKGAGADVIVSDQFPAGLRVLVVDDDITCLRLLEKMLRRCLYHVT
TCSQATAALKLLRERKGCFDVVLSDVHMPDMDGFKLLELVGLEMDLPVIMMSADGRTSAVMRGI
SHGACDYLIKPIREEELKNIWQHVIRKKWNENKEQEHSGSFEDNDRHKRGNDAENASSVSEGAEG
VLKGQKKRSIAKDEDDAELENDDPSASKKPRVVWSVELHQQFVSAVNHLGIDKAVPKRILELMN
VPGLTRENVASHLQKFRLYLKRLSGVAQQGGISNPFCGLLDSNVKLNPLGRFDIQVLAASGQIPPQ
TLAALHAELFGHPAGSMVTTVDQPALLQASVQGPKCIPAEHGMAFGQPLVKCQTNMSKHFPQNV
VSAEEVATGFVAWPSNSVGTVGPISNLGGMSSKNSNMLMDVVQQQKQQQQSPQLPSLPEPSRIIN
VQPSCLVVPSQSSATFQAGNSPASVNQNCSYSRSPMIDYSLLSPQSNNSSLNIGQISNGDLKTTGVS

GYSGSGSISPLSSCSVNPDNTSQQGQSSTMTIRAARQLPGLGPNISNFQGSYGAKSSEVLDQGPLRN
LGFVGKCTSIPSRLAVDEFESTMSNLEHEKLYMETNGNKVKQEPNSNAVKQEPNVEFMDNAKVLI
PILPQLPPNTSDLMSVFKE

>PtRR16 | Type-B | Gene ID: 7459182
MAALQRVASSLSTSASSYGSCKGAGADLMFSDQFPAGLRVLVVDDDITCLRLLEKMLCRCLYNV
TTCSQATAALKLLRERKGCFDVVLSDVHMPDMDGFKLLELVGLEMDLPVIMMSADGRTSAVMR
GIRHGACDYLIKPIREEELKNIWQHVIRKKWNENKEQEHSGSFEDNDWYKRGNDDTENASSVNEG
AEGVLKGQKKRNIAKEEDDNELEIDDPSASKKPRVVWSVELHQQFVSAVNQLGIDKAVPKRILEL
MNVPGLTRENVASHLQKFRLYLKRLSGVAQQGGISNTFCGPLDSNVKVNPLGRFDIQALAASSQIP
PQTLAALHAELFGRPTGSLVTTVDQPALLQASMQGPKCIPVEHGVAFGQPLVKCQTNISKHFPRNV
ISVEDVASGFGAWPSNSVGTVGSSSNLGGMNSQNSNMLMDILQQQKQQQQPLQQSSLSEPSRSIN
VQPSCLVVPSQSPATFQAGNSPASVDQNCSYNRSTMIDYSLLSAQSNNSSLNIGQILNGDLKTTGVS
GYSGSVSISPLSPCSVDPDNTSQQGRSSTMAIRAVRQLPGLVPNISNLQGSYSARSSEVLDQRPLRN
LGFVAKCTSIPSRLAVDEFESTMSSLDHEKSYMEISGNKVKQEPNIEYMDNARVGIPILQQLPPAPS
DLMSVFTE

>PtRR17 | Type-B | Gene ID: 7482119
MASTQSSNALSSSLPKIHILIVDDDSTSLSVVSATLKTVSYKDNRNLELIVVTVKKPFDALSILRLKK
GLFDLVVSDLHMPEMNGMELQKQVEEEFKLPVIIMSSDESKNVISRSLEGGAAFYIVKPANKVDLK
NVWHYAVAIKTGNSLSIKEIEGSREPSYSSTLVERLSLEDVNSATSINDEKRYRKNGRKEERKSTKD
DQEVDSQPASKKPKVVWTNSLHNRFLLALNHIGLDKAVPKRILECMSVRGLSRENIASHLQKYRIF
LKKVAERGGCSSKNLSGRDDLRTNFAPSQPFMMFKNVQQEYSQRVTRASIQPGLGGNTIPALGGS
SFRNLHSPNQQAPSNNSILQSPYGQSRLFGNANSANQANQIRSGLESNYSIGENMPSYGGVIPNGLT
HGTRPMQMYHPQNQARSYLQNFGSPPLINRFGAVGIQATNYGSGVGDIGIINNSLNTNNNYVGIRV
TTDGRLIGSGQMQLNTNELSNGFSDGGHPCLMNWARNGNMNVASIGFNIASENYVARRGSFSAG
FEGANQFFPPTPFTSVYHGNSTPMLLPPPPPQHQLGLGNGGQNDFVFGLMNNSPPILGGNSNQQPQI
GEGEPNVSDMLLEPTNNPPAYQLPQGVAQMSTPLHELLTPDFLNSLHIDDNTPRNEQPSSQVQSGE
GAMNPICGSDANSMDGYFPNFDQQNGNGDLVDSELNNPQPVDNGPTGNQAWDDDLLESLFGK

>PtRR18 | Type-B | Gene ID: 7479549
MMAVDDQRGGNSVNEKKFPVGMRILAVDDDPICLKVLENLLRKCLYEVTTTNQAVTALEMLRE
NRNKYDLVISDVNMPDMDGFKLLELVGLEMDLPVIMLSSHGDKEFVYKGVTHGAVDYLLKPVR
MEELKNIWQHVIRRKKWYPEDQNRSPNQDKGDDGAGEGEQGVTSTGSADQNGKLNRKRKDQD
DEDEEEGEDGDDNEVSGNQKKPRVVWSVDLHQKFVAAVNQMGLDKAVPKKILDLMNVDGLTR
ENVASHLQKFRLYLKRLSSGGNQQGNMVSAFGAKDSSYLQMSSVDGFGDFRSMHGPGRLSTTSL
SSYPSGTFLGRLNSPGGLTLQGIASSGLHQPGLLPTNQGTNLFQGISSALEPKQLQMKSINHIGEFNQ
KGDTAGFTLASCFPDVKVTIGSLSNTISGATSSPPILQANPQQNHNRGSLVTQSSLSMPSLNQESFD
VGVQGSSNFLNHSRCDDNWQGAVQLSTFPSLPIGEPFRHDQLPSSTLRGNISSTTCHIVNSPIDFSST
GSLTAPLEDSRVDMQGQTDFIGNIFQNTTNYISKNRWEENSQNYNPGLNGSFGAMNSLVSGNGSS
GALNQSMDQRKRFGASMIGQSNSGTLSMFQHPDVENFALDPKMRSNEDVILAQTKSQNGFVQNN
YESLDDIMNEMIKQVREYFFCFIHLNYELHMRSFQLFQSPFQVFVL

>PtRR19 | Type-B | Gene ID: 7489463
MTVEDQRGCNSVNEKMFPVGMRVLAVDDDPICLKVLENLLRKCQYEVTTTNQAVTALEMLREN
RNKYDLVISDVNMPDMDGFKLLELVGLEMDLPVIMLSSHGDKEFVFKGITHGAVDYLLKPVRLEE
LKNIWQHVIRRKKWYPQDQNGSPDQDKGGDGAGEGEQVTSTGSADQNGKVNRKRKDQDEEEEG
EGEDGNDNEESGNQKKPRVVWSVELHQKFVSAVNQLGLDKAVPKKILDLMNVDGLTRENVASH
LQKFRLYLKRLSCGANQQPNMVAAFGAKDSSYLRMGSLDGFGDFRSVHGPGQLSTTSLSSYPPGS
LLGRLNSPGGLTLQGIASPGLLQPGHSRSLNNPLNTLGKLQPGLLQTNRGSTNLFQGISSALDPKQF
QLKSMNHTGDFNHKGDATSFTLAGCFPDVKVTIGSLGNTISSAANNPQMLHVNPQQNQTRRSLAT
QSSLSMPSLNQESFDVGVRGSSNFLDHSRCDDNWQGVVQMSTFPSNSLPLGEPFRHDPLPTSTRRD
NISSTTSHIVNNPLDFSSSGSLTAPLEDSRLDMQGQADLVGNIFHNTNYTSKNRWGENSQNFNPCL
NGSFGAMNSLVSGNGSMNPLSQSMDQRKRFDASVLGQSNSGTLSMFQHLEAGNSALDPTGMRIP
KMRSNEDFLLEQTKSPNGFVQNNYDSLDDIVNAMIKRV

>PtRR20 | Type-B | Gene ID: 7476782
MASSQSSNIIQNSSLLKIKILVVDDDSTSLSIVSAMLKTCSYKENRKLELFVVTVKNPFDALSTLRLK
KGLFDLVVTDLHMPEMNGMELQQQVDEEFKLPVIIMSSDDSEKVILRTLEGGAAFYIVKPINKDDL
KNVWQYAVATKTGKYSLSIKEIGGSQESSSSTVFDQKISVDEVSSAKSTNIKICNEKDRINKNRKRT
KEDQEVDSQLAPKKPKVVWTNSLHSRFLQAINHIGLDKAVPKRILEFMSVPGLSRENVASHLQKY
RIFLKKVAERGTSSSKNLSGRALKSNFASSQPSLMLKNFQQEYSQFSRQQQVLRASVQAGFGGNTI
PTPDGGSNFGSLHSPNQQAPNNNSALQSPYKQSRSFGNPTGFQQLSFGNANQANKKRSGLESNNN
TGTNPPASRESVPNGLTHGKSPMQIYQPQNQGRSNLQSFDSSEHNKFGSIGVQATNYRSGVGNIGN
NNNSLNTNNNYAGIRVNTDGQLIGAGQLQVSKNKLSNGFGNGGNCGLMNWTHNGNMNVASSSY
LAQRGSFPASIESANQFPPTLFTSTVYQGNTPMLLPPPPPPPQQQQQLGLGNGGQHDFAFGLVNNSP
PIFGGNYNQQQQFGQGELNFSDMLLEPTNNLTAHQPQGVDQVSTPLHDLLHPDFLNSLHIDDNTP
WNEQPSSQVQSGEEAMNPFFGGNASSMDGYFPNFNQQKENGDLMDSELSSLYPDDNGQTGNQV
WDDDFLESLFGNESLPMD

>PtRR21 | Type-B | Gene ID: 7477058
MENGFSSPRNDSFPAGLRVLVVDDDPTWLKILEKMLKRCSYEVTTCGLARDALNLLRERKGGYDI
VISDVYMPDMDGFKLLEHVGLEMDLPVIMMSVDGETSRVMKGVQHGACDYLLKPIRMKELRNI
WQHVFRKKIHEVRDIEILEGIESFQMTRNGSDLYEDGHFLCGEDLTLIKKRKDIESKHDEKDIGDNT
SAKKARVVWSVELHQKFVKAVNQIGFDKVGPKKILDLMNVPRLTRENVASHLQKYRLYLSRLQK
GNDFNNPVGMIKQSDSPLSDSAGSFGSQNSINLQPTDVSNGCYGFSVSSLVLHNVDPRSHNGDRKI
LVTTPVAEPKRGVTVDAPNPRKPRISQMEYGHPLAPHFNDGFSHLPMPGQKQLTRADYPQPARAIS
SAHSLTEREIGCPVKIKPSRNDYGSNASHVSSTANAVDSIPLQTKTNSTNHQVSSTSSMENQGLNM
NCLTDVESSRKNINLGMPPFAPLDEDLQVRFVPGDYYMNLGLQNIEVPEYFDPSLLTDVPIHLNDG
LRFDYEFYDPTEYSLIDQSLFI

>PtRR22 | Type-B | Gene ID: 7458409
MTVEQGIGDSNIDQFPIGMRVLAVDDDPTCLLLLETLLRRCQYNVTTTSQAITALRMLRENKNKFD
LVISDVHMPDMDGFKLLELVGLEMDLPVIMLSANGDPKLVMKGITHGACYYLLKPVRIEELKTIW
QHVIRRKKSDNKDRNSSDNRDKPNQGSSEAVPDQKLNKKRKDQNGDEDEDHDEDEDEHENEDPT
TQKKPRVVWSVELHRKFVAAVNQLGVDKAVPKKILDLMNVEKLTRENVASHLQKYRHYLKRIST
VANQQANMVAALGSSDASYLQMNSMSVLGLHSLAGSVQFHSTPFRSLQSSGMLDRLNSPAVLGI
HGLPSPGVIQLGHVQTAPHTANGLSHFQPVGHRGNNGNILQGMPMPLELDQIQSNKGVNYIPELPT
HLDDTASFPVSSGSTDMKIIAGSSNSPFVGVSNKHLMLEGHGQGLQDGQKSGKQSSLSAGSLNPG
YSSHFPDHGRCNDNWSNAVPSNGAQSDSFTLNDYFKQSTLHPSAIRDRMSTMALQSRNNPCDVSS
VSTLPMHLQDSKADLPCRVGVATVSSNAGQLINNGSLGWDDRRQDDPYHSNGLSNSINSAFPING
NGSPNGFNLDPNNLFFQRTTSFISTGPSNFVDTSLMKHNEVECSAMETLVRSKDGYLLGQEKPQDS
SVSNNFGSLEDLVSVMINQPTILG
