# Supplementary material for: Genome-Wide Identification and Expression Analysis of Cytokinin Response Regulator (RR) Genes in the Woody Plant Jatropha curcas and Functional Analysis of JcRR12 in Arabidopsis
Source: Int J Mol Sci. 2022 Sep 27;23(19):11388. doi: 10.3390/ijms231911388 (PMC9570446; doi:10.3390/ijms231911388)
Supplement: Supplementary file 1 [file ijms-23-11388-s001.zip › Text S4. Predicted amino acid sequences for the 22 rice response regulators (RRs).pdf]

Supplementary Text S4 Predicted amino acid sequences for the 22 rice response regulators (RRs)

>OsRR1 | Type-A | Gene ID: 4335937
MEGGRGVTRVLLVDDSPVDRRVVQLLLSSSACAGSFHVIAVDSAKKAMEFLGLKEEGKEQAIDM
VLTDYCMPEMTGYELLKAIKALSPLKPIPVIVMSSENEPQRISRCMNAGAEDFIVKPLQSKDVQRL
RNCSPANTQCCDAGSDGKPPLLLLPSDHVVVDATAASPPPPPSRRRAHFAGVAMVLHSSSVELSH
YFPFLFKFILLVYAILCLGELLHRWSNGCFLNLWCA

>OsRR2 | Type-A | Gene ID: 4329677
MGAEAVRVLVVDDSPVDRRVVELLLRAHCGGGGGAAAGEAAPFHVTAVDSGKKAMELLGRRR
GDRDHLTPSSPAAAAAANDQAIDIVLTDYCMPEMTGYDLLKAIKALGSPNPIPVVVMSSENEPQRI
SRCLTAGAEDFILKPLKMNDVQRLRKCSGATRPKSAVAGDDDRCCNTAKKAAAAAAATPEQQQ
QQQRSSHLAGLAMVMNASSFEVSHYFQLIFKLILLAYAVLCLSQLLHRWSNGSSLLSLWCA

>OsRR3 | Type-A | Gene ID: 4331245
MSTKTVPEPEPHVLAVDDSIVDRTVISRLLRSSKYRVTTVDSGKRALEVLSLDRNVHMIITDYCMP
EMTGFDLLKRVKESAELKEIPVVLMSSENSPTRIRRCLEEGAEDFLIKPVRPSDVSRLCNRVIMK

>OsRR4 | Type-A | Gene ID: 4325747
MTVVDAESRFHVLAVDDSLIDRKLIEMLLKNSSYQVTTVDSGSKALELLGLRDEGDDSSSSPSSSSP
DHQEIDVNLIITDYCMPGMTGYDLLKRVKGSSSLKDIPVVIMSSENVPARINRCLEDGAEEFFLKPV
KLADMKKLKSHLLKRKQQLPMAAAAPDKPPHKPDEAAASAAAIAEAATAQTDGIISDCSCSGSSK
RKAAAMEQEVISSPDQRTKPRLSSTSSGLAVET

>OsRR5 | Type-A | Gene ID: 4336439
MATCRSRGVERGGAPHVLAVDDSSVDRAVISGILRSSQFRVTAVDSGKRALELLGSVSLRHPLPFC
CWLRKCGGVFCLRGRKRFFFFGSQEPNVSMIITDYWMPEMTGYELLKKVKESSRLKEIPVVIMSSE
NVSTRINRCLEEGAEDFLLKPVQPSDVSRLCSRVLR

>OsRR6 | Type-A | Gene ID: 4337372
MAAAAQAPAAAKVVVATSPRAGGGGGGGGDRKVVPVVVAAAAGDEAQSEMHVLAVDDSSVD
RAVIAKILRSSKYRVTTVESATRALELLCLGLVPNVNMIITDYWMPGMTGYELLKRVKESSQLKEI
PVVIMSSENVPNRISRCLEEGAEDFLLKPVRPSDVSRLCSRIR"

>OsRR7 | Type-A | Gene ID: 107276271
MEMRVPAAVTGGCGCGVDGGGGCCRGGGKLADWEEGKDDEMKSVVVKGWTRMAQVVPLHD
NASAEDDDDDEEDDDEDDDDDDDEDDEEEAAPPYVMAVDDSSVDRAVITALLRRSKYRVTAVD
SGKRALEILGSEPNVSMIITDYWMPEMTGYDLLKKIKESSELKQIPVVIMSSENVPTRISRKMLCFFS
LLVSDAWKKARRTSCSSPSDRRTSPA

>OsRR8 | Type-A | Gene ID: 107275331
MSSPHVLVVDDTLVDRHVVSMALMRHNVRVTAVESVMQALMFLDSEHDVNMIVSDYCMPDMT
GYDLLMEVKKSPKLAHLPVVIASSDNIPERIRKCLDGGAKDYILKPVKIVDLPRILNYI

>OsRR9 | Type-A | Gene ID: 4349747
MAVAIEAPFHVLAVDDSLPDRKLIERLLKTSSFQVTTVDSGSKALEFLGLHDHEDSPISTQSDQQEV
AVNLIITDYCMPGMTGYDLLKKIKESSYLRDIPVVIMSSDNIPSRINRCLEEGADEFFLKPVRLSDMS
KLKPHILKSRCKEHYQQEQNLQSNSESNNSSNPTSENSSSSTSSNSHKRKAVDEEILPHTIRPRHS

>OsRR10 | Type-A | Gene ID: 4351462
MAVAIEAPFHVLAVDDSLPDRKLIERLLKTSSFQVTTVDSGSKALEFLGLHDHEDSPISTQSDQQEV
GVNLIITDYCMPGMTGYDLLKKIKESSYLRDIPVVIMSSDNIPSRINRCLEEGADEFFLKPVRLSDMS
KLKPHILKSRCKEHYQQEQHLQSNSESNNSSNPTSENSSSSTSTNSHKRKAVDEEILPHTIRPRHS

>OsRR11 | Type-A | Gene ID: 4330059
MSSIGAGAGGAVVGAAVAAVAVGGGAPPHVLAVDDSSVDRAVIAGILRSSRFRVTAVDSGKRAL
ELLGSEPNVSMIITDY

>OsRR12 | Type-A | Gene ID: 9270123
MSSPHVLVVDDTHVDRHVISMALMRHNVRVTAVESVMQALVFLDSEHDVNMIVSDYCMPEMTG
YDLLMEVKKSPRLVHLPVIIASSDNIPERIRKCFDGGAKDYILKPVKIADVPRILNYI

>OsRR13 | Type-A | Gene ID: 4335184
MAFQTQGSNLRALLVEDIKVNRMILSQMLRKFQVETTVVQNGKEAVELFLGGETFDIVLTDNLMP
IMTGPEAISKIRAMGATDVMIVGVSVDANSMEEFKDAGADLCVPKLKLEILEHILQETRSKKNKSS
A

>OsRR14 | Type-A | Gene ID: 4334066
MARKMIRVLLVEDEEINRVVARAALKAAGGGDVVDEAENGEVAVQRVRDAAAPYDLVLMDKQ
MPVMDGHEATRRIRGMGVTTPIVAVSSDGLPADVDAFITAGADDFTSKPLSKEKLGVILAKFRLA

>OsRR15 | Type-A | Gene ID: 107275387
MSSPHVLVVDDTHVDRHVVSMALMRHNVRVTAVESVMQALMFLDSEHDVDMIVSDYCMPDMT
GYNLLMEVKKSPKLAHLPVVIASSDNIPERIRKCLDGGAKDYILKPVKIVDVPRIMKYI

>OsRR16 | Type-B | Gene ID: 4325022
MDATAFPYGLRVLVVDDDPTWLKILEKMLRKCSYEVTTCGLARVALDILRERKNKFDIVISDVNM
PDMDGFKLLEHIGLEMDLPVIMMSIDGETSRVMKGVQHGACDYLLKPVRMKELRNIWQHVYRK
KMHEVKEIEGNDSCDDLQILRNSFEGLDEKSLFMRSDSDTMRKRKDVDKDHADQESSDGNTVKK
ARVVWSVDLHQKFVNAVNQIGFDKVGPKKILDLMNVPGLTRENVASHLQKYRLYLSRLQKQNEE
RILGAARQDFSHKGTSENLNLRSSFQEQPSNIANGYPHASQNIQTQANMLDSQLEDTKSTVPLPVP
DKKRTLASDAADSQNVTSASSLGGVLSFKSMPVNQDRKPSETMILECQAWTGGIPSKQFMQYPKH
NHERCDLLGDYSCLPKPDLEHPVGPSNLYAPPPLISMSCGMEGDARDFSDVKPAIMDCIKSLSPAL
TCTVDSVSVQLSDSVVTSIDGDLKSSGVDGLPSIKDCCLDQTNSQGSLRPSQEPSIIGSTELASLPED
LPSYPLHGVSLENIGLSSIDLLNYSDAMILSGLQSNWYDDLEFSSEMMDYPSIDECLFASS

>OsRR17 | Type-B | Gene ID: 4328516
MTVEERQGRVGGHGVSGGGGGRDQFPVGMRVLAVDDDPTCLKILENLLLRCQYHVTTTGQAAT
ALKLLRENKDQFDLVISDVHMPDMDGFKLLELVGLEMDLPVIMLSANGETQTVMKGITHGACDY
LLKPVRLEQLRTIWQHVIRRKNCDAKNRGNDDDAGQKAQGMNNEGESIGANRNKRQSRKSRDE
NGDDGDDSDENSNENGDSSTQKKPRVVWSVELHRKFVAAVNQLGIEKAVPKKILDLMNVENITR
ENVASHLQKYRLYLKRLSTDASRQANLAAAFGGRNPAYINMNSFGNYNAYGRYRTVPTAGHTQ
ANNILTRMNSPSAFGVHGLLHSQPIQLGHAQNNLSTSLNDLGGLNNGNMIRGAQMSTILTGPSGNS
FPNISNGAPLATANRSLQPLESSNQQHLSRVHSSSADPFSTLVGESPQFPDLGRTTNTWQTAVPSNI
QDRGHNDNMSQATLHMNGPKIEPVSSFTSSNQIPLLGNEMQGQVASLASNVPIAFNQDTSPFNYGS
STNSRDMLNNSHVFSNSSINTSLPNLSLDNPAVPRQTLDRGNTGIVSPMQDGRIHHQAVSNQLNYN
DDLMRTTGLQRGLSGGLDDIVVDMFRPDREDDGVPYIDGDWELV

>OsRR18 | Type-B | Gene ID: 4331008
MRAAEERKGVVPAARRRDQFPVGMRVLAVDDDPVCLKVLETLLLRCQYHVTTTNQAAIALKML
RENRDMFDLVISDVHMPDMDGFKLLELVGLEMDLPVIMLSVNGETKTVLKGITHGACDYLLKPV
RIEELRNIWQHVIRRKFSTRDRANLDFYEECNKPPNADSDHVHGHVTCGSPDQSGRPSKKRKEYCS
EEEDEGEVNTQDIDDPSAPKKPRVVWSVELHRKFVAAVNQLGIDKAVPKRILELMNVEKLTRENV
ASHLQKYRLYLKRLSAVASQQVSIVAALGGRDPFLHMGGFEGLQGYQAFTSSAALSSFTPHGLLN
SPRNNPAALGTQGVPASKSIQTMSGSHTLSHSINDANKYHLSLPGNQKGNLGQGLATSLGQTQMQ
QKWIHEETDDLSTILSGNGLSNGMSGTLQSVTSSPLLPQELAECTQAKIVSQPSIRTSSVSSEHIEGA
VGVSSGLLESRVSQQSTIPLSGFSANGLLIHGSFNNTCANKLGGTSSSCAPARSSNDLMVARDTKG
GASSFGGAMLLPPDTEQKYLNFGGGNGLKQKFDDRTADSLFDLKFVWSSVPSSQLASNIGAHHA
MSQRWNNSSSNSSNIGARMIGQATSSGSTVIPQMKTDFLVSGDMAMPKNASDLSIPKLQSELSSSS
CSFDGLLNSIVKVEKDDVTFSDDLGCGDFYSLGACI

>OsRR19 | Type-B | Gene ID: 4332111
MAPVEDGGGVEFPVGMKVLVVDDDPTCLAVLKRMLLECRYDATTCSQATRALTMLRENRRGFD
VIISDVHMPDMDGFRLLELVGLEMDLPVIMMSADSRTDIVMKGIKHGACDYLIKPVRMEELKNIW
QHVIRKKFNENKEHEHSGSLDDTDRTRPTNNDNEYASSANDGAEGSWKSQKKKRDKDDDDGELE
SGDPSSTSKKPRVVWSVELHQQFVNAVNHLGIDKAVPKKILELMNVPGLTRENVASHLQKFRLYL
KRIAQHHAGIANPFCPPASSGKVGSLGGLDFQALAASGQIPPQALAALQDELLGRPTNSLVLPGRD
QSSLRLAAVKGNKPHGEREIAFGQPIYKCQNNAYGAFPQSSPAVGGMPSFSAWPNNKLGMADST
GTLGGMSNSQNSNIVLHELQQQPDAMLSGTLHSLDVKPSGIVMPSQSLNTFSASEGLSPNQNTLMI
PAQSSGFLAAMPPSMKHEPVLATSQPSSSLLGGIDLVNQASTSQPLISAHGGGNLSGLVNRNPNVV
PSQGISTFHTPNNPYLVSPNSMGMGSKQPPGVLKTENSDALNHSYGYLGGSNPPMDSGLLSSQSKN
TQFGLLGQDDITGSWSPLPNVDSYGNTVGLSHPGSSSSSFQSSNVALGKLPDQGRGKNHGFVGKG
TCIPSRFAVDEIESPTNNLSHSIGSSGDIMSPDIFGFSGQM

>OsRR20 | Type-B | Gene ID: 4340320
MLLGALRMEERKGLMGRERDQFPVGMRVLAVDDDPVCLKVLETLLRRCQYHVTSTNQAITALK
LLRENRDMFDLVISDVHMPDMDGFKLLELVGLEMDLPVIMLSVNGETKTVMKGITHGACDYLLK
PVRIEELRNIWQHVVRRKFGNRERNNLDFSKECNKPQSADTDHGPYQPTCGSSDQNGRSSRKRKE
LHGEDDDEGDDNDYQENDEPSAAKKPRVVWSVELHRKFVAAVNQLGIDKAVPKRILELMNVEK
LTRENVASHLQKYRLYLKRLGAVASQQASIVAAFGGRDPSFLHIGAFEGLQSYQPFAPSAALPSFN
PHGLLTRTSAAAAFGLQELAAPSSTIQTSTGNVTVGHCLEENQQANLAQGLTAAIGQPQLQQNWI
HQEGNGLSDVFSGSSLTNTLSSTLQRVPSSSLPPQELLECKQAKVSMPPSIRIPPSSSALLERTLGVST
NLGDSSISQQGALPIDGGFSADRLPLHSSFDGAVATKLDTSLAASQREIGQQGKFSVSMLVSPSDNL
ALAKNAKTGASSSGSTIILPLDTARHSDYLQFGGASNSLQKMDGQKQDHIQSSNIIWSSMPSTQLPS
DTQIHNTQNQRLDSGSFNHNIGAHLADQTNASASILPQMKFDTRISEEKMKQKNTYDLGSSKLQG
GFNSSGCNFDGLLNSIIKVEKDDLPFMDNELGCDLFPLGACI

>OsRR21 | Type-B | Gene ID: 4341665
MAATQATAARKFPEGLRVLAVDDSPVCLMLLEALLRRCKYQPTMTRDAATALRMLRERPGDFDL
VISDVHMLDMDGFKLLELIGLEMDLPVIMQSANGELETMMKGVTHGACDYLVKPVSLKDIQNIW
QHVWRKRKLDIRNHNGGYNDGGELVGATRTKRKYTRKMRNDGDNYGENKENMDSTLKRQRVV
WTPELHRDFVIAVHELGVDRAVPRKILRMMKVDYMTRENIASHLQKYRLYLKRISTQTGMDPDQ
FPEKWKYMNELDALKNYCENGRYRLTPAIASSSSSNPFARMNSASALATNGFLPTHSVQLKNSQR
NMAMGTVGHGGSPGNNPVFQPLQNSSNARKCFPSGPSGSSFANISNGLVLDTDDSGSSYAGMFCK
SMWETSNGSPSCHSGNSSANKSNNGVSAPANQFQVQSKFGFSALANQFPVQSNCGFSAPANQYQ
VQSNGGFSVPANQFPVQSNGEFLAPTNQFPVQYPEVNNQPLVQMNQSSTNHFSTIGNDYQFPDLA
NCSKYWQPTAPSMFPDLGHNDGTSFRPSQANIANINQLSSFAASSGQEPMFGDELHGQMSPIMSTI
SLSDFDDQMGSFNIGNDTSPAEMMHDNFSLGSDSNISSSTPTDSSFGSTFPDFHLDSPEMPAQMLNG
GDEDGILLPVLDDTVDQQDLFDQLDENNGREKLGSGRCVRKGPFECFF

>OsRR22 | Type-B | Gene ID: 107276289
MDHRELWPYGLRVLVIDDDCSYLSVMEDLLLKCSYKVTTYKNVREAVPFILDNPQIVDLVISDAFF
PTEDGLLILQEVTSKFGIPTVIMASSGDTNTVMKYVANGAFDFLLKPVRIEELSNIWQHIFRKQMQD
HKNNNMVGNLEKPGHPPSILAMARATPATTRSTATEASLAPLENEVRDDMVNYNGEITDIRDLGK
SRLTWTTQLHRQFIAAVNHLGEDKAVPKKILGIMKVKHLTREQVASHLQKYRMQLKKSIPTTSKH
GATLSSTALDKTQDHPSRSQYFNQDGCKEIMDYSLPRDDLSSGSECMLEELNDYSSEGFQDFRWD
SDKQEYGPCFWNF
