# Supplementary figures and images for: Genome-Wide Identification and Expression Analysis of Cytokinin Response Regulator (RR) Genes in the Woody Plant Jatropha curcas and Functional Analysis of JcRR12 in Arabidopsis
Source: Int J Mol Sci. 2022 Sep 27;23(19):11388. doi: 10.3390/ijms231911388 (PMC9570446; doi:10.3390/ijms231911388)

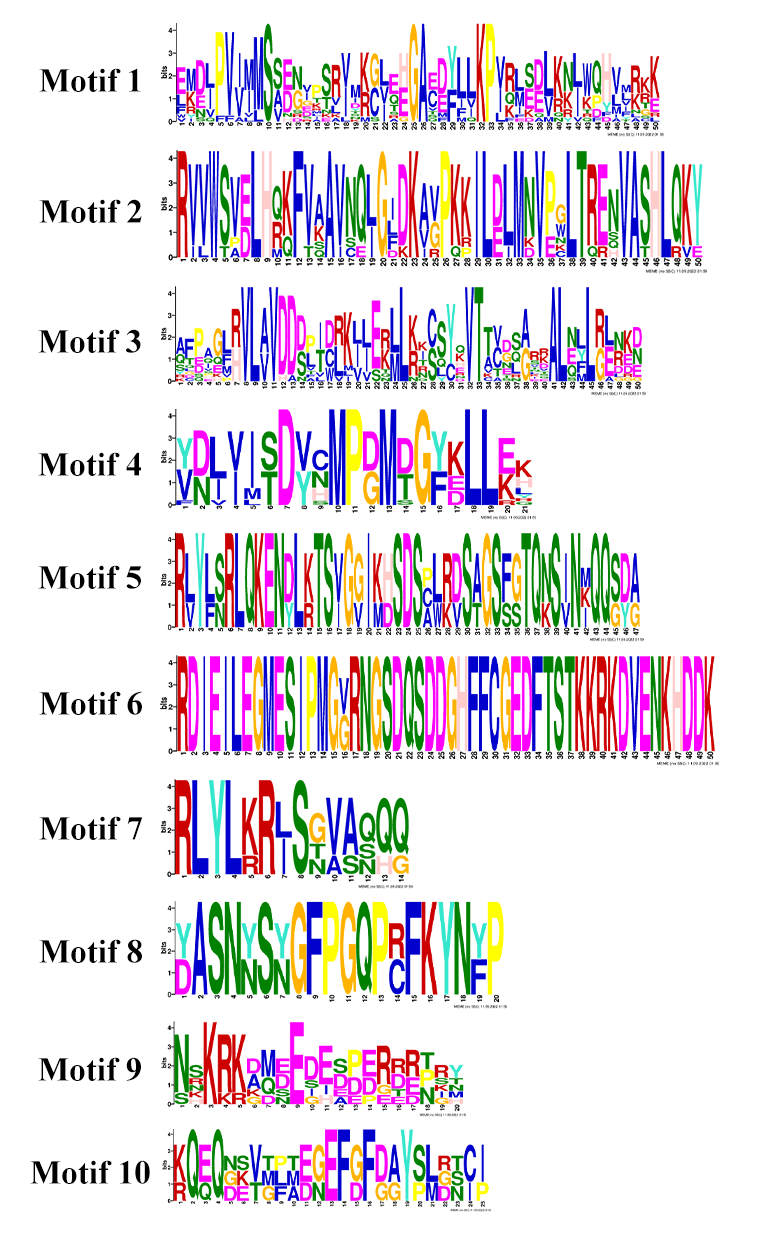

Supplement: Supplementary file 1 [file ijms-23-11388-s001.zip › Figure S1. Sequence logos of the motifs in RR proteins.tif]
